# Psychological response of pregnant women during COVID-19 pandemic lockdown

**DOI:** 10.1590/1980-220X-REEUSP-2023-0088en

**Published:** 2023-12-22

**Authors:** Laura Lasso-Olayo, Dominika Pasierb, Víctor Berdejo, Ángel Gasch-Gallén

**Affiliations:** 1 Universidad de Zaragoza, Facultad de Ciencias de la Salud, Departamento de Fisiatría y Enfermería, Zaragoza, Spain.; 2 University of Rzeszow, Institute of Health Sciences, College of Medical Sciences, Rzeszow, Poland.; 3 Universidad de Zaragoza, Facultad de Ciencias, Departamento de Física Aplicada, Zaragoza, Spain.

**Keywords:** Anxiety, Pregnancy, Perinatal Care, COVID-19, Pandemics, Ansiedad, Embarazo, Assistência Perinatal, COVID-19, Pandemias

## Abstract

**Objective:**

In this study we explore anxiety and fear of COVID-19 in women in the process of pregnancy during lockdown due to the SARS CoV-2 pandemic and their relationship with sociodemographic and clinical factors in a tertiary referral level hospital.

**Method:**

A descriptive cross-sectional study was carried out in pregnant women at the Lozano Blesa University Clinical Hospital in Zaragoza (Spain), from April 15, 2020 to May 15, 2020. A total of 168 women was recruited when they went to the hospital for a routine blood test for pregnancy. They answered a sociodemographic and clinical data questionnaire, the Spielberg state-trait anxiety questionnaire for anxiety, and a visual analog scale for fear of COVID.

**Results:**

Frequency of Trait anxiety was 50.7% (95% CI: 42.7–58.7) and 52.7% (95% CI: 44.7–60.7) for State anxiety. The mean visual analog scale for fear of COVID-19 in relation to pregnancy was 57.2 (95% CI: 52.4-61.8). A positive correlation was found between the scales. Statistically significant differences were found between rural and urban areas.

**Conclusion:**

The emotional impact of the COVID-19 is high among pregnant women and the levels of anxiety are higher than usual in these groups of women during the pandemic lockdown.

## RESUMEN

**Objetivo:**

En este estudio exploramos la ansiedad y el miedo al COVID-19 en mujeres en proceso de embarazo durante el confinamiento por la pandemia de SARS CoV-2 y su relación con factores sociodemográficos y clínicos en un hospital de tercer nivel de referencia.

**Método:**

Se realizó un estudio descriptivo transversal en mujeres embarazadas en el Hospital Clínico Universitario Lozano Blesa de Zaragoza (España), desde el 15 de abril de 2020 al 15 de mayo de 2020. Se reclutó a un total de 168 mujeres cuando fueron al hospital para un análisis de sangre de rutina para control durante el embarazo. Respondieron un cuestionario de datos sociodemográficos y clínicos, el cuestionario de ansiedad estado-rasgo de Spielberg para la ansiedad y una escala visual analógica para el miedo a la COVID.

**Resultados:**

La frecuencia de ansiedad rasgo fue del 50,7% (IC 95%: 42,7-58,7) y del 52,7% (IC 95%: 44,7-60,7) para la ansiedad estado. La media de la escala analógica visual de miedo a la COVID-19 en relación al embarazo fue de 57,2 (IC 95%: 52,4-61,8). Se encontró una correlación positiva entre las escalas. Se encontraron diferencias estadísticamente significativas entre las zonas rurales y urbanas.

**Conclusión:**

El impacto emocional de la COVID-19 es alto entre las mujeres embarazadas y los niveles de ansiedad son más altos de lo habitual en estos grupos de mujeres durante el confinamiento pandémico.

## INTRODUCTION

The new SARS-CoV-2 coronavirus was first detected in Wuhan (Hubei province, China) in December 2019, spreading at first in China and then worldwide. On March 11, 2020 the World Health Organization (WHO) declared COVID-19 a pandemic^(1)^. There was an increase in cases and deaths, with 767 million confirmed cases worldwide as of July 5, 2023, and mortality rate exceeding 6.9 million^(2)^. In Spain, there were 256 deaths per 100,000 inhabitants until June 30, 2023^(3)^.

On March 14, 2020, the Spanish government declared a state of national alert and as of March 16, lockdown was imposed, where the entire population was made to stay at home for more than three months, except for activities considered essential. For the first time in this century, a global pandemic has occurred due to a virus, generating great uncertainty about the consequences that this may have on the mental health of pregnant women, who are a high-risk population.

An association has been found between outbreaks of infectious diseases and psychological symptoms such as anxiety or other mental illness^(4)^. Furthermore, the level of stress and anxiety can also increase during outbreaks of infectious diseases^(5)^. Studies indicated that pregnancy and postpartum are vulnerable times to relapse into a mental illness if it exists, or for it to debut^(6)^. Depression and anxiety are the most prevalent psychiatric disorders during pregnancy and postpartum in a normal situation^(7)^; therefore, a situation of global pandemic and lockdown may exacerbate them^(5,8)^.

It has been observed that elevated levels of cortisol associated with anxiety, depression, and stress in the mother can be transferred to the fetus through the placenta^(9,10)^. All abovementioned factors are associated with adverse consequences in the mother and newborns^(8)^ and motor and behavioral disturbances in early childhood^(11)^.

The prevalence of anxiety disorder during pregnancy is 15.2%^(12)^. Additional global pandemic situation with a national health alert and lockdown may result in greater susceptibility of pregnant women to anxiety and fear. Adequate quality of care in the process of pregnancy, delivery, and postpartum need to be maintained in a pandemic situation^(13)^.

So far, a large number of studies focus their attention on the physical symptoms of infected women, giving little relevance to maternal mental health^(14)^.

There are very few studies available on the psychological consequences of lockdown in a pandemic for pregnant women^(5,8)^. This study aimed to evaluate anxiety and fear of Covid-19 in pregnant women and its relationship with sociodemographic and clinical factors during the Covid-19 outbreak, in a tertiary level referral hospital.

## METHOD

### Design of Study

Descriptive cross-sectional study aimed at evaluating the psychological impact of the COVID-19 epidemic on pregnant women. This study was designed following a checklist^(15)^.

### Local

It was carried out at the Hospital Clínico Universitario Lozano Blesa in Zaragoza (Spain) during the COVID-19 epidemic. The Spanish authorities imposed a strict confinement from March 15 to May 4, 2020, with an order to stay at home for all non-essential workers and ban on outdoor activities.

### Population

The study population is pregnant women during COVID-19 pandemic lockdown. Sample high- and low-risk pregnant women who attended the Central Laboratory Unit of the Hospital to be screened for gestational diabetes or have the oral glucose tolerance test (OGTT) to determine glucose levels were selected to participate in the study, regardless of gestational age and parity. The recruitment time was selected purposefully, since gestational diabetes screening is a routine test performed in the entire pregnant population to monitor their pregnancy; therefore, it was possible to access them during lockdown, when many appointments were carried out via telemedicine.

The minimum sample size required was estimated at 115 subjects using a specific tool^(16)^ and the data provided by the National Institute of Statistics of Spain^(17)^. In total, 168 pregnant women consented to participate and 150 completed questionnaires fully. After obtaining written informed consent from the selected participants, the women were asked to complete the questionnaire, designed ad hoc, based on previous studies on the subject^(5)^. Our hospital policies and procedures were followed to prevent the spread of SARS - CoV2 infection, including the use of hand sanitizer and the distribution of sanitary gloves.

### Data Collection

Data collection was carried out from April 15, 2020 to May 15, 2020 by the principal researcher. The pregnant women filled in socio-demographic and clinical data related to their pregnancy and their anxiety level was measured with the Spielberg state-trait anxiety questionnaire (STAI). Moreover, the women were asked to complete the visual analog scale (VAS) that reflected fear of the Covid-19.

The pregnant women filled in sociodemographic data regarding their age, educational level, income level, marital status, number of children, religion, home ownership, nationality, and area of residence.

The women reported the date of their last period, the estimated date of delivery, whether the current pregnancy was a single or multiple pregnancy, the existence of pathology in the current pregnancy, previous pregnancies and previous types of births, the medical and surgical history.

The STAI is a 40-item self-reported scale for state (STAIs) and trait (STAIt) anxiety^(18)^. This instrument is an inventory created to assess anxiety as a state (momentary, transitory) and anxiety as a trait (as a more stable condition). It is made up of 40 items divided into 2 subscales: trait and state, with likert-type responses from 0 to 3. It can be used in the normal population or in patients. For its correction, a state anxiety score and a trait anxiety score are obtained, adding each of the items of each subscale, so that they can be used separately. The questionnaire is self-administered. It is the most commonly used scale to assess anxiety, and has been validated for use in women during pregnancy. The higher the score, the greater the anxiety^(8,19)^. Both the STAI-S scale and the STAI-T scale can score between 20 and 80. Based on other studies, the cut-off point was established at 39, indicating the existence of anxiety^(8)^. Its psychometric properties are extensively detailed in the bibliography^(18)^.

The women were also asked to complete the visual analog scale (VAS) for fear of COVID-19, which is intuitive and generally easy to apply^(20)^. This ranged from 0 (no fear at all) to 100 (extreme fear) and referred to the following question: *How much fear do you feel regarding the coronavirus pandemic in relation to your pregnancy?*

### Data Analysis

The categorical variables were described using absolute and relative frequencies and measures of central tendency (mean, median) and dispersion (standard deviation, interquartile range) were calculated for numerical variables, which allowed the characterization of the studied population. For non-normal quantitative variables, the median and the 25th and 75th percentiles (P25 and P75, respectively) were studied.

To determine the dependence between two categorical variables, the χ^2^ statistical test was applied. To determine differences between the groups, a Mann-Whitney test was applied. To determine the relationship between two non-normal quantitative variables or scores, the Spearman correlation coefficient (Rho) and its p-value were calculated. The level of significance was set at p ≤ 0.05, and the minimum power for the different tests used with our sample size was established at 0.8. The IBM SPSS software 20.0^(21)^ was used for statistical analysis, with the exception of the calculation of the power of the different tests employed for our sample sizes, which were performed using the statistical programming language R.

### Ethical Aspects

This study was approved on April 29, 2020 by the Clinical Research Ethics Committee of Aragon with identification code PI20/189. There are no conflicts of interest.

In addition, in order not to create an ethical vacuum, the women who obtained high scores in anxiety were referred for evaluation and follow-up by the health service.

## RESULTS

A total of 168 women was enrolled in the study; of them, 150 women, aged 16-45, were included in the study. The mean age was 32.7 (SD: 5.9).

As it can be seen in Table 1, most of the participants (n = 90) had secondary education (60%), 64 women (42.7%) earned up to 1000 EUR per month; 127 women (84.7%) were married or had a partner; 77 women (51.3%) considered themselves as non-believers. The majority of studied women (n = 102; 68%) were Spanish; 83 (55.3%) rented a flat. Most of them, 117 (78%), did not present any type of pathology in their current pregnancy. Forty-one women (27.3%) gave natural birth in previous pregnancies. Eighty-seven (58%) lived in urban areas while 60 (40%) in rural areas. At the time of the study, 18 women (12%) were in the first trimester of pregnancy, 40 (26.7%) in the second trimester, while 92 (61.3%) were in the third one. We can see that some of the variables were not a total N of 150. This is because some women did not answer these and it was calculated in the statistical analysis.

**Table 1:** Socio-demographic and clinical variables of the Sample– Saragossa, Aragon, Spain, 2020.

	N	%
EDUCATION		
Up to secondary	90	60
Higher	60	40
INCOME		
Up to 1000 EUR	64	42.7
1000-1500 EUR	45	30
More than 1500 EUR	41	27.3
MARITAL STATUS		
Married/cohabitating partner	127	84.7
Single/other	22	14.7
RELIGION		
Catholic	42	28
Non-believer	77	51.3
Other	31	20.7
NATIONALITY		
Spanish	102	68
Other	37	24.7
HOUSING TYPE		
Own or with family	67	44.7
Rental	83	55.3
PATHOLOGY IN CURRENT PREGNANCY		
Yes	27	18
No	117	78
TYPE OF PREVIOUS BIRTHS		
Natural	41	27.3
Caesarean section	19	12.7
Instrumental	10	6.7
AREA OF RESIDENCE		
Urban	87	58
Rural	60	40
TRIMESTER		
1st	18	12
2nd	40	26.7
3rd	92	61.3

The mean STAI-S score was 39.1 (SD: 10.4) while STAI-T was 39.9 (SD: 9.0). The mean VAS score for fear of COVID-19 in relation to pregnancy situation was 57.2 (SD: 28.0).

As it can be seen in Table 2, positive correlation was observed between the scales, although it is moderate in the case of VAS. The trend indicated that anxiety increases with perceived fear of COVID-19 in relation to pregnancy.

**Table 2:** Characteristics of STAI-T and STAI-S, VAS fear of COVID-19 in the sample and correlations among scales – Saragossa, Aragon, Spain, 2020.

	STAI-S	STAI-T	VAS fear of COVID-19
Min-Max	20-70	25-67	0-100
Mean (SD)	39.1(10.4)	39.9(9.0)	57.2(28.0)
CI 95%	37.1-40.5	38.4-41.4	52.4-61.8
STAI-S	1.000	0.677^*^	0.335^*^
STAI-T	0.677^*^	1.000	0.358^*^
VAS fear of COVID-19	0.335^*^	0.358^*^	1.000

^*^The correlation is significant at the 0.01 level (bilateral).

Of the study group, 77 women (51.3%) presented clinical anxiety; 56% presented anxiety in the first trimester; 55% in the second trimester; while 49% in the third trimester.

Clinical variables have been correlated with anxiety levels looking for statistical significance. No statistically significant correlation was found between maternal age, state anxiety, and fear (p = 0.53 and p = 0.97). However, a statistically significant correlation was found between maternal age and state anxiety (p = 0.01). Spearman’s correlation coefficient shows that it is positive and weak (Rho = 0.22).

As seen in Table 3, an association was found between anxiety and the area of residence. The urban residents obtained higher STAI-T scores (55.2%), p = 0.024. Similarly, the urban residents were more afraid of COVID-19 in relation to their pregnancy (59.8%), p = 0.025. However, no significant differences were found in terms of the socio-demographic and clinical variables: marital status, income, nationality, housing type, pathology in the current pregnancy, type of previous births, or gestation trimester. In the case of the religion item in VAS, it showed trend of p = 0.069.

**Table 3:** Relationship between STAI-S and STAI-T and socio-demographic and clinical variables in the sample – Saragossa, Aragon, Spain, 2020.

	High STAI-S N (%)	High STAI-T N (%)	VAS N (%)
EDUCATION	42 (46.7)	43 (47.8)	46 (51.1)
Up to secondary	32 (53.3)	28 (46.7)	32 (53.3)
Higher	p = 0.424	p = 0.894	p = 0.790
INCOME	29 (45.3)	37 (57.8)	34 (53.1)
Up to 1000 EUR	26 (57.8)	20 (44.4)	25 (55.6)
1000-1500 EUR	24 (52.2)	14 (34.1)	19 (46.3)
More than 1500 EUR	p = 0.432	p = 0.054	p = 0.675
MARITAL STATUS	62 (48.8)	70 (55.1)	67 (52.8)
Married/cohabitating partner	11 (50)	9 (40.9)	11 (50)
Single/other	p = 0.919	p = 0.218	p = 0.811
RELIGION	22 (52.4)	17(40.5)	17 (40.5)
Catholic	38 (49.4)	39 (50.6)	47 (61)
Non-believer	14 (45.2)	15 (48.4)	14 (45.2)
Other	p = 0.830	p = 0.564	p = 0.069
NATIONALITY	56 (54.9)	49 (48)	54 (52.9)
Spanish	14 (37.8)	17 (45.9)	19 (51.4)
Other	p = 0.075	p = 0.827	p = 0.868
HOUSING TYPE	37 (55.2)	31 (46.3)	33 (49.3)
Own or with family	37 (44.6)	40 (48.2)	45 (54.2)
Rental	p = 0.195	p = 0.814	p = 0.545
PATHOLOGY IN CURRENT PREGNANCY	15 (55.6)	15 (55.6)	14 (51.9)
Yes	56 (47.9)	52 (44.4)	62 (53)
No	p = 0.471	p = 0.297	p = 0.915
TYPE OF PREVIOUS BIRTHS	24 (58.5)	23 (56.1)	23 (56.1)
Natural	9 (47.4)	9 (47.4)	9 (47.4)
Caesarean section	3 (30)	2 (20)	5 (50)
Instrumental	p = 0.247	p = 0.122	p = 0.804
AREA OF RESIDENCE	45 (51.7)	48 (55.2)	52 (59.8)
Urban	29 (46.0)	23 (36.5)	26 (41.3)
Rural	p = 0.491	**p = 0.024**	**p = 0.025**
TRIMESTER	10 (55.6)	9 (50)	8 (44.4)
1st	21 (52.5)	22 (55)	21 (52.5)
2nd	43 (46.7)	40 (43.5)	49 (53.3)
3rd	p = 0.709	p = 0.462	p = 0.789

**Figure 1:**
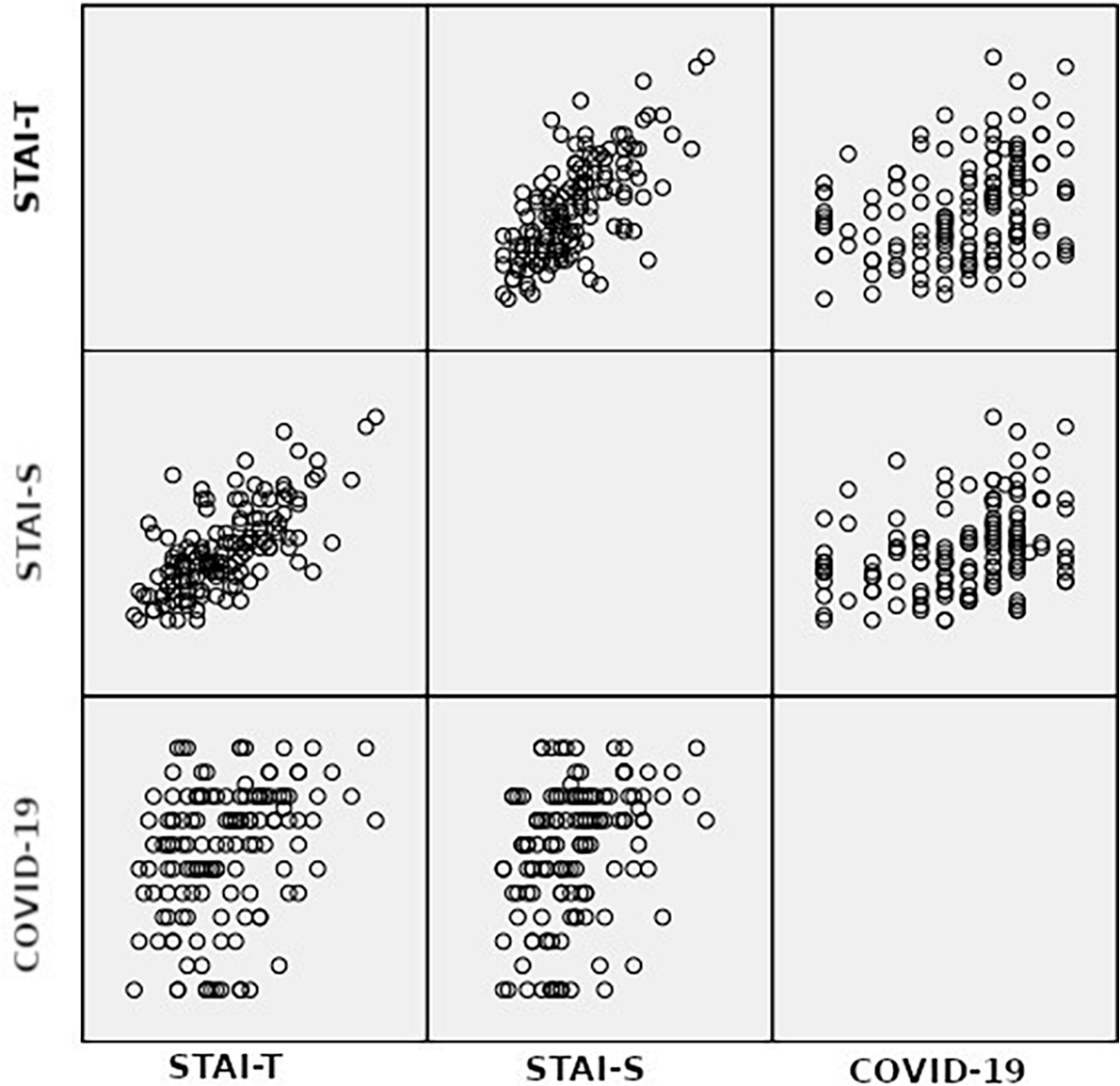
A scatter plot matrix for STAI-S, STAI-T and fear of COVID-19.

Figure 1 presents accumulation of data of each variable in relation to the others. Our results show adequate reliability of the elements of the STAI scale for our sample.

As Table 4 presents, statistically significant differences were found between the median values for both STAI-S and STAI-T and the perception of fear of coronavirus.

**Table 4:** Relationship between anxiety and the fear of the coronavirus in relation to pregnancy – Saragossa, Aragon, Spain, 2020.

	Low fear COVID-19	High fear COVID-19	p-value
STAI-S: Median (IR^**^)	14 (13)	23 (16)	<0.001^*^
STAI-T Median (IR^**^)	15.50 (10)	22 (14)	<0.001^*^

^*^Mann-Whitney U test for independent samples

^**^width of the interquartile range

Women who were most afraid of COVID-19 in relation to their pregnancy presented higher levels of anxiety both in STAI-S and STAI-T.

## DISCUSSION

This study aimed to evaluate the anxiety and fear of COVID-19 of pregnant women in Spain during the COVID-19 and analyze its association with socio-demographic and clinical variables. This being a high-risk population due to its potential impact on mental health.

The work we have carried out indicates that in situations of stress related to an infectious pandemic, anxiety increases. These consequences have also been observed in 2003 in the SARS pandemic^(22)^ and in 2015-2016 with the Zika virus^(23)^.

However, not many studies have been conducted on the mental health status of pregnant women during lockdown^(5,8,24,25)^.

It is especially important to carry out studies that analyze the prevalence of anxiety during pregnancy and to follow up these women during the postpartum period, especially in times of pandemic in which anxiety in the general population is already triggered^(26)^. The high levels of anxiety are consistent with previous studies in a population similar to ours^(5,8,25)^.

In the first three months of pregnancy, higher levels of anxiety in the context of a COVID-19 pandemic^(5)^ have been observed in other studies. In our study, similar results were observed in women in the first trimester of gestation, who were the ones with the highest levels of STAI-S (55.6%).

Of the study group, 77 women (51.3%) presented clinical anxiety; 56% presented anxiety in the first trimester, 55% in the second trimester, while 49% in the third trimester. Our results were in line with those found in other studies^(5,8)^.

In relation to maternal age, we found that there was no statistically significant correlation or it was weak with respect to the study variables.

Statistically significant differences were found between rural and urban areas. These data were not published before in any study. The lower values in the VAS to measure the fear of COVID-19 in relation to their pregnancy presented by women from rural areas may be due to the fact that rural women avoid cities overcrowding, minimizing their exposure to the virus. Furthermore, in the rural environment, having relationship with neighbors is more common, which may influence a feeling of having control that perhaps is not achieved in the urban environment.

The impact of the pandemic on maternal mental health needs to be further evaluated, as there has been some resistance to giving it the importance it deserves^(24)^.

Our findings may be useful to design interventions to help the pregnant population in their mental health in times of pandemic. In addition, due to the high levels of anxiety detected in the pregnant population, we consider that these results suggest the need to implement a screening to determine the level of anxiety during pregnancy follow-up.

The limitation of the study was the single-centered study design. Another limitation could be that the women answered the questions in the hospital setting, since being in a hospital during a pandemic is likely to be a stressful event.

However, the trends observed suggest that in the context of the health crisis caused by Covid-19, the impact on anxiety is high, as well as its relationship with fear of the pandemic, regardless of age, social class, socioeconomic and academic level.

## CONCLUSION

The emotional impact of the COVID-19 outbreak is high among pregnant women and the levels of anxiety are higher than usual in these groups of women.

Statistically significant differences were found between rural and urban areas, which may suggest that the rural environment becomes a protective factor against the high perception of fear in relation to the COVID-19 situation in relation to pregnancy. The fact that no statistical significance was observed between anxiety and some of the sociodemographic and clinical characteristics in our study population led us to the conclusion that pandemics do not differentiate between social class, socioeconomic or academic level, but rather affects all of them equally. This makes the effect of the pandemic itself more devastating, since the prevention programs developed must refer to all of them equally, not being able to isolate a group at greater risk.
